# Casein Functionalization Using High-Pressure Homogenization and Emulsifying Salts

**DOI:** 10.3390/polym17070931

**Published:** 2025-03-29

**Authors:** Anthony Fuchs, Danielle Stroinski, Ashley Gruman, Grace Lewis

**Affiliations:** Department of Animal and Food Science, University of Wisconsin—River Falls, River Falls, WI 54022, USAdrs6321@psu.edu (D.S.); alg6488@psu.edu (A.G.)

**Keywords:** casein, high-pressure homogenization, emulsifying salts, foaming, emulsions, caffeine, encapsulation

## Abstract

In milk, casein proteins orientate themselves into spherical micellar structures with hydrophobic casein subtypes concentrated in the core, while hydrophilic casein subtypes populate the exterior. Previous research demonstrated that milk with the addition of emulsifying salts coupled with high-pressure homogenization induced an unprecedented amount of casein micelle dissociation. This research aims to quantify the extent of casein micelle dissociation in diluted skim milk and evaluate the functionality of these proteins following emulsifying salt treatment coupled with high-pressure homogenization. To evaluate the extent of micellar dissociation, dilute skim milk solutions (20% *v*/*v*) were prepared with a varying amount of treatment: no processing (control), just emulsifying salts (Treatment E, 100 mM sodium hexametaphosphate), just high-pressure homogenization (Treatment H, at 300 MPa), and EH (a combination of E and H treatments). Samples were then put through varying filter sizes (0.22 µm, 0.05 µm), and the permeates were analyzed using sodium dodecyl sulfate-polyacrylamide gel electrophoresis. In the control group (20% skim milk), 9.35% ± 2.53% casein protein permeated through a 0.05 µm filter. Alternatively, 93.2% ± 7.71% casein protein was present in EH samples post-filtration through a 0.05 µm filter, demonstrating a significant processing-induced dissociation of casein micelles. A potential benefit to this casein micelle size reduction is the exposure of highly functional hydrophobic subunits from the core of the micelle. In agreement, compared to the control samples, the EH samples had higher foam expansion index values (138.3% ± 12.58% vs. 33.33% ± 14.43% at 0 h), foam stability (113.3% ± 5.774% vs. 21.67% ± 2.887% after 8 h), emulsifying activity (ca. two-fold higher), and interaction with caffeine. These data demonstrate that E, coupled with H, enhances skim milk system functionality, and these changes are likely due to micellar dissociation and protein conformational changes. This work has direct applications in dairy systems (e.g., dairy foams, dairy ingredients) as well as implications for potential processing strategies for other protein-rich systems.

## 1. Introduction

Casein proteins, at ca. 80% of milk protein content, are the most abundant proteins in milk. These proteins are generally described as calcium-binding phosphoproteins, and they consist of four peptides: α_s1_, α_s2_, β, and κ-caseins at approximate ratios of 4:1:3.5:1.5, respectively, in bovine milk [1]. These peptides have a propensity to bind calcium at phosphate centers and self-associate through a combination of electrostatic, hydrogen, and hydrophobic interactions, leading to the formation of casein micelles with typical sizes between 80 and 400 nm. Although considered to be relatively disorganized and polydisperse colloidal structures with over 20,000 individual protein molecules, it is generally agreed that casein micelles have highly hydrated, porous cores primarily composed of the α- and β-caseins and outer layers primarily composed of amphiphilic, glycosylated κ-casein [1,2].

Casein micelles are naturally stable against typical dairy manufacturing processes, including heat treatment (up to 100 °C) and homogenization (up to 100 MPa) [3]. Casein micelles have also received significant attention due to their ability to complex with polysaccharides [4], destabilize with action from specific proteolytic enzymes, aggregate upon sufficient acidification, and carry nutritionally significant molecules [3]. With this, they are highly functional in numerous dairy systems such as yogurt and cheese, but they have also been evaluated as potential nanocarriers for targeted drug delivery [5], nutraceutical protection [6], and other pharmaceutical applications. However, in their natural structure, casein micelles have shielded hydrophobic residues, limiting their potential as surface active agents as well as their drug loading capacity.

Several technologies have been investigated to enhance casein functionality, and several of these techniques operate by inducing casein micelle dissociation to expose internal protein residues [7]. Dynamic high-pressure technologies, including high-pressure homogenization (HPH) and high-pressure jet (HPJ) technologies, have shown some promise in manipulating the micellar structure at sufficient pressures (>100 MPa) [8]. Roach and Harte [8] identified a distinct, environment-dependent decrease in micellar size at HPH treatments exceeding 100 MPa followed by aggregation at pressures > 200 MPa. In a separate study, Roach et al. [9] found an increase in triclosan (i.e., a widely used and hydrophobic antimicrobial agent) association with casein proteins post-HPH and ethanol-induced micellar dissociation. With HPJ treatment ≥ 400 MPa, Hettiarachchi et al. [10] found pressure-induced micellar alterations enhanced skim milk functional properties, including foam expansion, foam stability, emulsifying activity, and viscosity. An alternative processing strategy to induce micellar dissociation includes the addition of emulsifying or calcium-sequestering salts (ES). The addition of these salts has been shown to chelate calcium from the core of the casein micelle, diminishing the micellar stability caused by calcium phosphate linkages and causing substantial dissociation [11]. ES, an approved food additive (21CFR133.169), are commonly employed in processed cheeses to create a homogeneous paste with enhanced meltability and stretchability.

The combination of these two processing approaches (HPH and ES) and the associated impact on system functionality has yet to be thoroughly evaluated. Our previous study [12] demonstrated extensive casein micelle dissociation and hydrophobic casein residue exposure with this combined processing approach (i.e., HPH and ES). The objective of the present work is to evaluate the functionality of this process-altered system, including foamability, emulsifying activity, and interaction with small hydrophobic molecules (i.e., caffeine). A processing-induced enhancement in functionality has direct applications in dairy systems (e.g., cappuccinos, ice cream) as well as potentially leading to new value-added applications for dairy proteins (e.g., encapsulation agents).

## 2. Materials and Methods

### 2.1. Sample Formulation and Ultra-High-Pressure Homogenization Treatments

To produce the control sample, pasteurized skim milk (SM; Kemps, El Paso, TX, USA) was combined with distilled water to achieve 20% SM *v*/*v* (i.e., 1:4 SM to water dilution, ca. 2% total solids). The initial SM had an average fat content of 0.07%, an average protein content of 3.76% (3.00% casein), an average lactose content of 4.91%, and an average total solids of 9.81% as determined by a calibrated MilkoScan FT2 (FOSS Analytics, Hilleroed, Denmark). SM was selected as a minimally processed, economically viable dairy stream with direct translation to industry applications. The associated dilution was consistent with our initial study [12] and allowed for the addition of ES without altering the concentration of milk components. Three treatment groups were then compared to this control sample as follows:

- Treatment E: 20% (*v*/*v*) SM samples with 100 mM sodium hexametaphosphate (SHMP);
- Treatment H: 20% (*v*/*v*) SM samples processed using HPH at 300 MPa;
- Treatment EH: 20% (*v*/*v*) SM samples with 100 mM SHMP and processed using HPH at 300 MPa.

Based on our previous study evaluating the impact of varying concentrations of SHMP and sodium citrate on dilute SM systems [12], the SHMP concentration (i.e., 100 mM) and HPH condition (i.e., 300 MPa) selected for this study were purposefully beyond critical limits determined to be sufficient for micellar dissociation ([SHMP] ≥ 1 mM, HPH ≥ 200 MPa). Additionally, of the processing levels evaluated, these processing levels provided the quantitative minimum in sample absorbance, reflecting a maximum in casein micelle dissociation. Although only SHMP was used in the present study with insight from our previous work [12], other ES vary in their critical concentration (i.e., the concentration that initiates casein micelle dissociation) [11], and the goal of this study was to provide insight into the functional impact of ES beyond critical concentrations and post-micelle dissociation.

For samples containing SHMP (Treatments E and EH), SM (at 20% *v*/*v*) was combined with distilled water and a SHMP stock solution (250 mM), in that order, to achieve a SHMP concentration of 100 mM (i.e., 1:2:2 SM to distilled water to SHMP stock, ca. 2% total solids). For samples undergoing HPH (Treatments H and EH), samples (20% *v*/*v* SM at 20 °C) were processed using a Nano DeBEE 45-2 high-pressure homogenizer (Pion Inc., Billercia, MA, USA) with a 0.13 mm nozzle. The unit was set to 300 MPa with a consistent back pressure of 6.9 MPa. Following pressurization, all samples were immediately cooled by an in-line heat exchanger with a co-current flow of water (20 °C), keeping the sample outlet temperature < 35 °C. Replicates (*n* = 3) were prepared and processed separately.

### 2.2. Sodium Dodecyl Sulfate-Polyacrylamide Gel Electrophoresis (SDS-PAGE)

Samples, after undergoing treatments, were put through varying filter sizes: 0.22 µm pore size nylon syringe filter (VWR, West Chester, PA, USA) and 0.05 µm pore size polytetrafluoroethylene syringe filter (Millex hydrophilic PTFE syringe filters, Millipore, Burlington, MA, USA). The permeate was collected, added to a reducing sample buffer (0.5 M Tris-HCl buffer, 10% sodium dodecyl sulfate, glycerol, 0.5% *w*/*v* bromophenol blue, and 2% *v*/*v* β-mercaptoethanol) at a 1:4 ratio of sample to buffer, and put into a boiling water bath for 5 min. Aliquots (10 µL) were then loaded into 12% TGX precast gels (Bio-Rad Laboratories, CA, USA). Gels were run at 200 V with a Tris/glycine/sodium dodecyl sulfate running buffer, stained for 1 h using Coomassie brilliant blue, and destained overnight. This procedure was modified from Oquendo et al. [13]. The density of protein bands was then evaluated using ImageJ software (Version 1.53t) [14]. The density of the permeate protein bands (i.e., filtered samples) was compared against unfiltered samples within the same gel, allowing for the calculation of residual protein post-filtration.

### 2.3. Emulsifying Activity and Emulsion Stability

Emulsifying activity was evaluated using a turbidimetric method modified from Hettiarachchi, Corzo-Martínez, Mohan, and Harte [10]. Commercial vegetable oil (Our Family, Grand Rapids, MI, USA) was combined with samples at a 1:9 ratio, and a coarse emulsion was formed using a rotary homogenizer (VWR 250 Homogenizer, VWR, Radnor, PA, USA) set at 10,000 rpm for 1 min. Sample aliquots (10 µL) were immediately transferred into 0.1% sodium dodecyl sulfate solutions (1.6 mL) and gently inverted three times. This solution was then transferred into a disposable cuvette, and absorbance was measured using a spectrophotometer (V-1200 Spectrophotometer, VWR, Radnor, PA, USA) with the wavelength set at 500 nm. Emulsifying activity was determined as follows:

EA = 2Td/ϕc(1)

where *EA* is the emulsifying activity (m^2^·g^−1^), *T* is the turbidity, which is 2.303 A/l with *A* = absorbance at 500 nm and *l* = path length (m), *d* is the dilution factor, *ϕ* is the discontinuous phase volume ratio, and *c* is the protein concentration (g·m^−3^). A separate aliquot was extracted from the initial emulsion after 30 min and evaluated following the procedure described above to reflect emulsion stability (EA_30_).

### 2.4. Foaming Capacity and Foam Stability

Sample aliquots (10 mL, 20 °C) were measured into disposable centrifuge tubes and vortexed at 3200 rpm for 1 min, following the method of Hettiarachchi, Corzo-Martínez, Mohan, and Harte [10]. The resulting foam height was recorded and compared to the initial liquid volume (10 mL) to determine a foam expansion index (FEI) as follows:

(2) $$FEI=[\frac{V_{0}-V_{liquid}}{V_{liquid}}]\times 100$$

where *V*_0_ is the initial volume of the sample measured immediately after 1 min of vigorous stirring (including any drained liquid), and *V_liquid_* is the liquid volume used to obtain the foam (i.e., 10 mL). Foams were then monitored for 8 h, and foam height was recorded at regular intervals during that period.

To evaluate foam stability in a cappuccino-like application, instant coffee (0.66 ± 0.02 g, Folgers Classic Roast, New Orleans, LA, USA) was added to undiluted, treated skim milk samples (100 mL, 20 °C) and frothed (Zulay Kitchen, Clearwater, FL, USA) for 5 s. Foam height was monitored for one hour.

### 2.5. Interaction with Caffeine

Sample intrinsic fluorescence (*λ_ex_* = 275 nm, *λ_em_* = 300–500 nm) was used to evaluate the extent of interaction between caffeine (CAS Number 58-08-2, Sigma Aldrich, Burlington, MA, USA) and hydrophobic protein residues (e.g., tryptophan) using a fluorescence spectrophotometer (Cary Eclipse, Agilent, Santa Clara, CA, USA). Based on a procedure from Gong et al. [15], samples were evaluated with and without caffeine (100 µM) addition, allowing for the development of a fluorescence profile pre- and post-caffeine addition. Samples were diluted with four distinct buffers, and these buffers were also used as blanks prior to sample measurements. For the dilutions without caffeine, samples (0.5 mL) were diluted with 3.5 mL buffer containing either (1) distilled water (control and H samples) or (2) 100 mM SHMP buffer (E and EH samples). For the dilutions with caffeine, samples (0.5 mL) were diluted with 3.5 mL of buffer containing either (3) distilled water with caffeine (control and H samples) or (4) 100 mM SHMP buffer with caffeine (E and EH samples). The final concentration of caffeine in all caffeine-containing samples was 100 µM.

### 2.6. Statistical Analysis

All procedures were completed in a randomized order with triplicate samples. Minitab software (v21.1.0, State College, PA, USA) was used to conduct a 1-way ANOVA with Tukey’s test applied for mean comparisons. The significance of treatment was considered at *p* < 0.05.

## 3. Results and Discussion

### 3.1. Processing-Induced Micellar Dissociation

**Figure 1:**
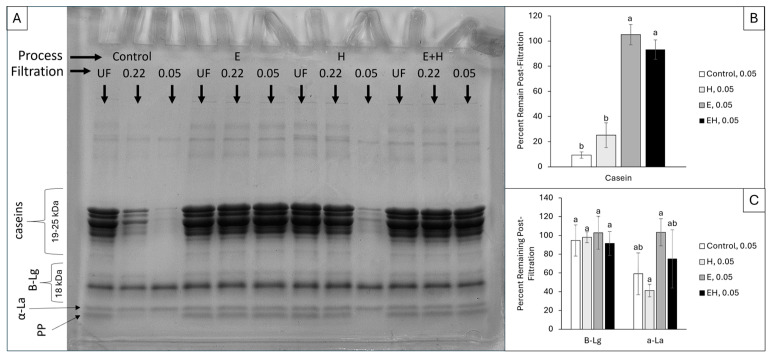
Sodium dodecyl sulfate-polyacrylamide gel electrophoresis (**A**) of milk system permeates following no filtration (UF), filtration through a 0.22 µm filter, and filtration through a 0.05 µm filter. The densities of the resulting protein bands were quantified using ImageJ. The samples filtered through a 0.05 µm filter were compared to the unfiltered (UF) sample, creating a percentage of protein remaining post-filtration for casein (**B**) and whey proteins (**C**). ^a,b^ superscripts reflect significant differences (*p* < 0.05) between samples within each protein group. Error bars reflect standard deviation (*n* = 3) of relative percentages within three distinct gels. Β-Lg = β-lactoglobulin, α-La = α-lactalbumin (ca. 14 kDa), PP = proteolysis products.

Casein micelle diameters typically range from 80 to 400 nm in bovine milk, with the smallest micelles existing at >50 nm [16]. SDS-PAGE was combined with a pre-filtration procedure to evaluate the extent of processing-induced casein micelle dissociation as well as monitor fluctuations in the micellar association for specific casein proteins. The smallest filter size used, with 50 nm pores, was selected to retain caseins associated within a typical micellar structure, allowing for only serum proteins (i.e., proteins no longer associated within micellar structures) to pass into the permeate. SDS-PAGE was conducted on the initial samples (i.e., control, E, H, and EH) and their associated permeates through two filter sizes (220 and 50 nm). A representative SDS-PAGE gel and the associated evaluation of protein band intensities are presented in Figure 1.

In the control sample (20% *v*/*v* SM), the permeate through the 220 nm filter contained 27.3 ± 16.8% of the casein proteins relative to the unfiltered sample, and the permeate through the 50 nm filter contained 9.4 ± 2.5% of the casein proteins relative to the unfiltered sample (Figure 1B). Following HPH (Treatment H), a larger population of casein proteins permeated through the 220 nm filter compared to the control sample, achieving 94.4 ± 3.6% casein proteins in the permeate post-filtration (*p* < 0.05). The addition of SHMP led to significantly (*p* < 0.05) more casein micelle dissociation in both the E and EH samples compared to the control sample, allowing 105.1 ± 8.1% and 93.2 ± 7.7% of the casein proteins to pass through the 50 nm filters, respectively. Fluctuations above 100% are likely due to the error associated with image analysis, as small differences in lighting can alter the measured densities; however, the intensity of the casein bands for the E and EH samples are visually comparable and not statistically different. There were no significant differences in the density of whey protein bands between samples and filtration procedures (Figure 1C).

As expected, intact casein micelles, such as those found in the control sample (20% *v*/*v* SM), were generally unable to permeate through the 50 nm filter. Upon exposure to HPH, there was some dissociation of casein micelles or reduction in casein micelle size, leading to enhanced permeation through the 220 nm filter in the H samples. In contrast, Roach and Harte [8] identified a sharp increase in casein micelle size and reduction in soluble casein proteins with HPH pressures ≥ 250 MPa applied to skim milk retentate. Differences in these findings could be due to the equipment used, milk system (skim milk vs. skim milk retentates), or integrity of the micelles due to the dilution buffer used (distilled water vs. Imidazole buffer). In the present work, the distilled water used for SM dilution modified the ionic equilibrium [17] which likely destabilized the micelles and caused increased susceptibility to HPH-induced dissociation relative to the study conducted by Roach and Harte [8].

With SHMP addition (100 mM), casein micelles were dissociated, allowing for complete permeation through 220 nm and 50 nm filters for both the E and EH samples. In agreement, previous work identified extensive micellar dissociation at the SHMP concentration used (100 mM), leading to a decrease in system opacity [11,12]. As this SHMP-induced dissociation was extensive, there was no differentiation in casein subtypes (α_s1_, α_s2_, β, and κ-caseins) in the permeate post-filtration.

The smallest protein band, identified as proteolysis products (PPs), is a result of the proteolytic activity of native enzymes in milk (e.g., plasmin) hydrolyzing milk proteins [18,19,20,21]. The PP band also had distinct intensity differences between samples, although the bands were too close to the whey protein bands to be accurately quantified using ImageJ. Specifically, there is a clear decrease in PP band intensity for the control and H samples post-filtration. Specifically, the PP content decreased for the control sample through the 220 nm filter, whereas the PP content decreased for the H sample through the 50 nm filter. However, when exposed to SHMP in E and EH samples, there was no longer a distinct density difference in these bands post-filtration.

It has been reported in other works that PPs naturally associate with the casein fraction in SM [19,22]. In the present study, the smallest filter (50 nm) removed the majority of the casein micelles in the control and H samples, thereby excluding any associated PP from the permeate as well. Upon SHMP addition, casein micelles were dissociated, releasing any PP associated with the micelles and allowing them to permeate through the smallest filter size (50 nm). The specific association between PPs and casein micelles cannot be determined without further experimentation, and there might also be some association between PPs and other proteins (e.g., bovine serum albumin, whey proteins). However, it can be speculated that PPs associate with larger proteins through a combination of hydrophobic interactions, calcium bridging, and electrostatic interactions due to their partial exposure upon HPH and complete exposure upon ES addition. Similar results were obtained by Di Marzo, Pranata, and Barbano [19], as they noted a large proportion (>88%) of PPs existed in a noncasein nitrogen precipitate (NCN) post-Kjeldahl method for protein determination, indicating the association of PPs with the casein fraction. This PP band has been reported to be γ_2_-casein and γ_3_-casein, which associate with the intact casein fraction due to their highly hydrophobic nature [22].

### 3.2. Processing-Induced Alterations in Skim Milk Foamability

**Figure 2:**
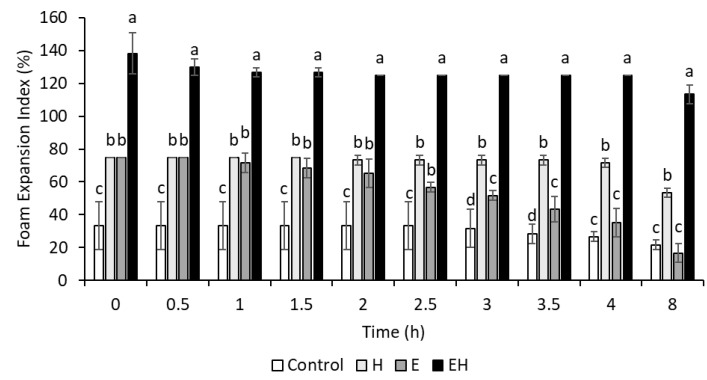
Foam expansion index (FEI) of milk systems (20% skim milk) through 8 h. E = milk system (20% skim milk) with 100 mM sodium hexametaphosphate, H = milk system (20% skim milk) processed using high-pressure homogenization at 300 MPa, EH = milk system (20% skim milk) containing 100 mM sodium hexametaphosphate and processed using high-pressure homogenization (300 MPa). ^a–d^ superscripts reflect significant differences (*p* < 0.05) between samples within one time period. Error bars represent standard deviation (*n* = 3).

**Figure 3:**
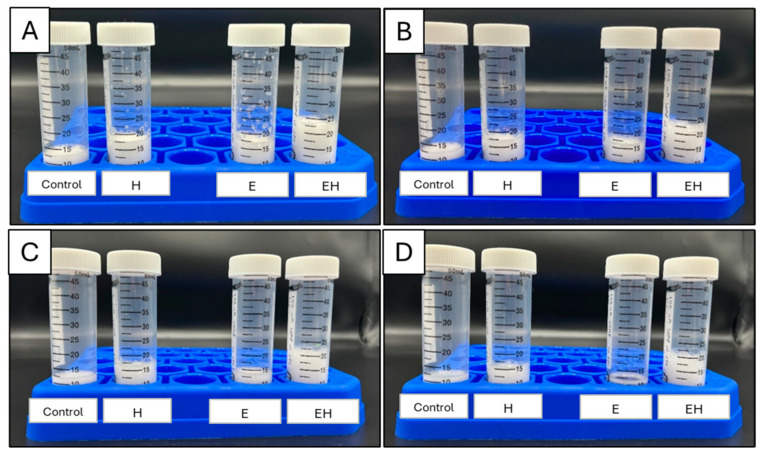
Images of milk systems (20% skim milk, 10 mL) post-foaming after 0 h (**A**), 2 h (**B**), 4 h (**C**), and 8 h (**D**). E = milk system (20% skim milk) with 100 mM sodium hexametaphosphate, H = milk system (20% skim milk) processed using high-pressure homogenization at 300 MPa, EH = milk system (20% skim milk) containing 100 mM sodium hexametaphosphate and processed using high-pressure homogenization (300 MPa).

It is generally agreed that dairy proteins support foam via a viscoelastic layer at the air–water interface. However, the functionality of casein micelles at air–water interfaces has been questioned, and some references suggest that individual caseins (e.g., β-casein) are more important in these cases [23]. With this, the extensive micellar dissociation achieved in the present work could aid in foam systems. To investigate this, the foam expansion index (FEI) and stability were evaluated and are presented in Figure 2. Images of the foams created are presented in Figure 3.

The initial FEI of the control sample (20% skim milk) was 33.3 ± 14.4% at 0 h. H and E samples behaved similarly in their initial foamability, both achieving FEI values of 75.0 ± 0.0% at 0 h. These values were significantly higher than the control sample (*p* < 0.05). In comparison, the EH sample had a significantly higher FEI (138.3 ± 12.6%) than all of the other samples at 0 h (*p* < 0.05).

Although the E and the H samples had similar initial FEIs, their foam stabilities were significantly different (*p* < 0.05). The H samples remained relatively stable through time, retaining a FEI of 53.3 ± 2.9% after 8 h, whereas the E samples were quite unstable, resulting in a FEI of 16.7 ± 5.8% after 8 h. The EH samples, having a combination of HPH application and SHMP addition, had a FEI of 113.3 ± 5.8% after 8 h, indicating a high foam stability.

The integrity of a foam relies on characteristics of both the foam interface and liquid lamella. Hydrophobic particles are thought to provide structural reinforcement to the foam interface [24], while hydrophilic particles retard foam drainage via a system jamming effect within the lamella [25]. Zhou, Yang, Sala, and Sagis [23] found that β-casein, with distinct hydrophobic and hydrophilic regions, was more likely to adsorb at interfaces than other casein subtypes. With this, the systems with extensively dissociated casein micelles (E and EH) and subsequently more exposed β-casein would logically have more units available to adsorb at the foam interface, causing enhanced foamability. However, the differences in foam stability between these treatments (E and EH) indicate that native β-casein alone might not be suitable for stabilizing foam interfaces in the long term, and these differences might be due to the liquid lamella. Chen et al. [26] found that foam stability improved with increasing concentrations of casein micelle aggregates present in the lamella. Although immediate filtration and SDS-PAGE of samples post-processing revealed extensive micellar disruption in E and EH samples, it is possible that HPH induced instability within the EH sample, allowing for some casein reaggregation to occur and exist within the liquid lamella. Additionally, it is also possible that HPH altered protein conformation after ES-induced micelle dissociation, allowing for superior interfacial properties for the EH samples relative to the E samples. There needs to be more research conducted to confirm the exact mechanism behind the observed foam stability. Regardless, the combination of ES addition and HPH allowed for superior foamability and foam stability.

**Figure 4:**
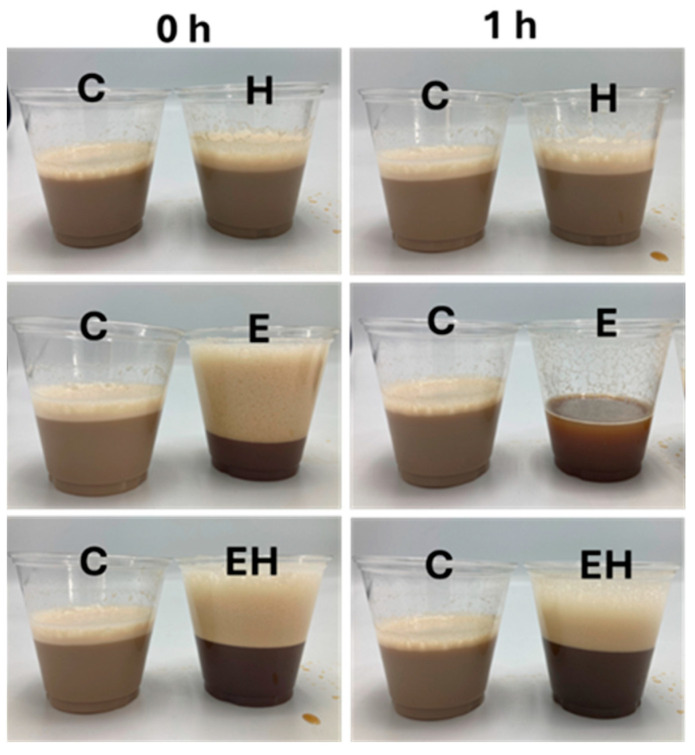
Application of milk systems in frothed coffee initially after frothing (images on the left) and after 1 h (images on the right). C = control milk system, E = milk system with 100 mM sodium hexametaphosphate, H = milk system processed using high-pressure homogenization at 300 MPa, EH = milk system containing 100 mM sodium hexametaphosphate and processed using high-pressure homogenization (300 MPa).

Within the dairy industry, there are numerous dairy-based foam applications, including cappuccinos, ice creams, and mousses, and dairy ingredients can be applied in other food categories (e.g., baked goods). For this SM system, a cappuccino-like application was evaluated and is presented in Figure 4. In agreement with the former results, frothed coffee sample E had a substantial initial foam, but this foam diminished in just 1 h. The frothed coffee sample EH had a high foamability and foam stability, allowing for the retention of a tall foam head beyond 1 h.

### 3.3. Processing-Induced Alterations in Skim Milk Emulsifying Properties

Like air–water interfaces, the ability of casein micelles to stabilize oil–water interfaces has been questioned and recently attributed to casein subunits, especially β-casein [23]. However, the properties of the continuous phase within an emulsion (e.g., viscosity, jamming particles) also impact emulsion characteristics. The present work evaluated a 10% oil emulsion in dilute milk systems (20% SM) after initial emulsification (EA_0_) and after 30 min (EA_30_), and the results are presented in Table 1.

Similar to the foam systems, the EH sample was most able to form and stabilize an emulsion with an EA_0_ of 61.12 ± 0.42 m^2^·g^−1^ and an EA_30_ of 36.52 ± 3.50 m^2^·g^−1^. These values are significantly higher than the other samples (*p* < 0.05). There were no significant differences between the EA_0_ values of the control, E, and H samples. Unlike in the foam system, H samples were not more capable of stabilizing an emulsion (EA_30_ = 20.24 ± 2.82 m^2^·g^−1^), yielding statistically similar results to the control after 30 min (*p* > 0.05). However, the E samples had enhanced emulsion stability after 30 min (EA_30_ = 28.36 ± 1.85 m^2^·g^−1^) compared to the control and H samples (*p* < 0.05).

For this emulsion system, the ES-induced dissociation of casein micelles and subsequent exposure of smaller casein units seemed to have the primary influence on emulsion stabilization as both E and EH samples had enhanced stability. Augusta Rolim Biasutti et al. [27] found that increased hydrolysis of casein improved emulsification capacity and suggested this could be due to an increase in protein contact points at the oil–water interface. However, these authors also found the benefit of protein hydrolysis was lost after 24 h of storage, at which time the extent of hydrolysis negatively impacted emulsion stability. With the noncontinuous phase (i.e., oil) occupying only 10% of the sample volume, the impact of particle jamming within the continuous phase was likely minimal, differentiating this phase from the foam system. However, with the enhanced emulsifying activity and stability identified in EH samples relative to other samples, the impact of micelle dissociation on emulsion properties was still enhanced by HPH, suggesting some HPH-induced protein conformational change that aids in interface stability as proposed in the foam system. Hettiarachchi et al. [28] found that condensed skim milk high-pressure jet-treated at 400 and 500 MPa had enhanced foaming and emulsifying properties compared to samples processed at lower pressures, and they suggested extensive pressure- and shear-induced casein micelle modifications caused these functionality alterations. Although the present system is processed using HPH under slightly lower pressures (300 MPa), it is possible that ES addition makes casein more susceptible to pressure-induced structure deformation. However, more research is needed to confirm this.

### 3.4. Interactions Between Caffeine and Amino Acid Residues Post-Processing

**Figure 5:**
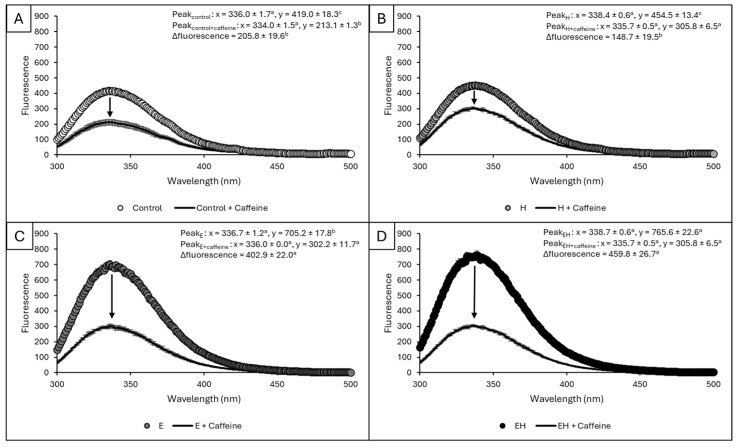
Fluorescence curves for control samples vs. control samples plus caffeine (**A**), H samples vs. H samples plus caffeine (**B**), E samples vs. E samples plus caffeine (**C**), and EH samples vs. EH samples plus caffeine (**D**). The x and y coordinates of the fluorescence peak for each sample are presented. The differences in peak height with and without caffeine addition for each treatment, as reflected by the arrows, are also presented within each graph as ∆fluorescence. ^a–c^ indicate significant differences between treatments (*p* < 0.05). Error bars represent standard deviation (*n* = 3).

The ability of protein residues to interact with small hydrophobic molecules (e.g., caffeine) has implications for encapsulation applications. To quantify these interactions, a fluorescence quenching method was used, evaluating any decrease in protein intrinsic fluorescence post-caffeine (100 µM) addition. The intrinsic fluorescence of a protein is related to the emission of relatively hydrophobic tryptophan and tyrosine residues at a defined excitation wavelength (280 nm). In aqueous environments, caffeine has complex hydration and limited solubility (1 g per 46 mL water) due to its heteroatomic bicyclic aromatic structure and propensity to self-associate via hydrophobic interactions [29]. In this case, caffeine is serving as a model for small (MW: 194.19 g/mol), relatively hydrophobic (LogP = −0.07) molecules [30]. The fluorescence curves for samples before and after caffeine addition are presented in Figure 5.

Regardless of the sample, there was no significant shifting of emission wavelengths as samples consistently achieved fluorescence maxima between 335 and 339 nm (*p* > 0.05). Prior to caffeine addition, the control samples and H samples had statistically similar intrinsic fluorescence values (419.0 ± 18.3 and 454.5 ± 13.4, respectively, *p* > 0.05), whereas the E samples had elevated intrinsic fluorescence values relative to these samples (705.2 ± 17.8, *p* < 0.05). The intrinsic fluorescence values for EH samples were significantly higher than all other samples (765.6 ± 22.6, *p* < 0.05). Upon caffeine addition, all samples experienced intrinsic fluorescence quenching, causing a reduction in emission intensity. This decline was statistically similar for control and H samples (*p* > 0.05), but ca. two-fold higher for E and EH samples (*p* < 0.05).

In the control SM, the addition of caffeine quenched some fluorescence, reflecting a natural ability for milk proteins to interact with target compounds. Milk proteins have previously been shown to have protective effects on other compounds, including anthocyanins [6,31], vitamin D [32], and β-carotene [33]. Caseins have been shown to have a greater protective effect than whey proteins [6]. Gong, Yang, Zhang, Yu, Gu, Li, and Wang [15] identified numerous binding sites between casein and anthocyanins from purple potato flour (two non-acylated and three acylated), causing associations via hydrogen bonds and van der Waals forces. However, there is a potential to enhance these interactions with processing techniques [9,32].

In the present study, upon ES-induced casein micelle dissociation, additional protein residues were exposed, yielding a higher initial intrinsic fluorescence in E and EH samples relative to control and H samples. These findings are in agreement with previous studies [12]. When caffeine was added to this dissociated system, it interacted with exposed protein residues, causing heightened fluorescence quenching relative to the control sample. To the best of our knowledge, the interaction between ES-dissociated casein systems and small compounds has not been previously investigated. The findings of this work suggest that these novel processing methods can be applied to enhance the encapsulation potential of casein in various food and pharmaceutical systems; however, additional research looking at specific interactions and system stability through time is required.

## 4. Conclusions

This study successfully demonstrated casein micelle dissociation and functionality enhancement due to coupled emulsifying salts and high-pressure homogenization treatment on dilute skim milk. Specifically, the combined processing approach (emulsifying salt addition and high-pressure homogenization, Treatment EH) yielded heightened foam expansion and stability, increased emulsifying potential and stability, and enhanced caffeine interaction relative to a control sample. In the majority of the tested applications (i.e., foamability, foam stability, emulsifying potential, and emulsifying stability), the functionality of EH samples exceeded samples treated with just emulsifying salts (Treatment E) and just high-pressure homogenization (Treatment H), demonstrating the novelty of this combined approach. Similar foam expansion and stability results for EH were observed in a cappuccino-like application, indicating potential applications for this system. Future work will evaluate specific compound encapsulations as well as the digestibility and gelation of these skim milk systems.

## Figures and Tables

**Table 1:** Initial emulsifying activity (EA_0_) and emulsifying activity after 30 min (EA_30_) of milk systems (20% skim milk) with 10% oil.

Sample *	EA_0_ (m^2^·g^−1^)	EA_30_ (m^2^·g^−1^)
Control	36.84 ± 4.26 ^b^	15.57 ± 1.96 ^c^
E	48.36 ± 8.31 ^b^	28.36 ± 1.85 ^b^
H	42.43 ± 1.65 ^b^	20.24 ± 2.82 ^c^
EH	61.12 ± 0.42 ^a^	36.52 ± 3.50 ^a^

* E = milk system (20% skim milk) with 100 mM sodium hexametaphosphate, H = milk system (20% skim milk) processed using high-pressure homogenization at 300 MPa, EH = milk system (20% skim milk) containing 100 mM sodium hexametaphosphate and processed using high-pressure homogenization (300 MPa). ^a–c^ superscripts represent significant differences (*p* < 0.05) within each column. Values are presented as mean ± standard deviation (*n* = 3).

## Data Availability

Data are contained within the article. Raw data will be available on request from the authors.

## Abbreviations

The following abbreviations are used in this manuscript:

HPH	High-pressure homogenization
HPJ	High-pressure jet
ES	Emulsifying salts
SHMP	Sodium hexametaphosphate
EA_0_	Initial emulsifying activity
EA_30_	Emulsifying activity after 30 min
FEI	Foam expansion index
CNPP	Casein proteolysis products
NCN	Noncasein nitrogen precipitate
SDS-PAGE	Sodium dodecyl sulfate-polyacrylamide gel electrophoresis
